# Spatiotemporal strategies that facilitate recruitment in a habitat specialist tree species

**DOI:** 10.1093/aobpla/plw033

**Published:** 2016-07-11

**Authors:** Shivani Krishna, Hema Somanathan

**Affiliations:** Indian Institute of Science Education and Research, CET Campus, Thiruvananthapuram 695016, India

**Keywords:** Janzen-Connell, microsite, *Myristica fatua*, Myristicaceae, Myristica swamps, nutmegs, temporal escape

## Abstract

Our understanding of processes underlying plant recruitment emerges from species and habitats that are widely distributed at regional and global scales. However, the applicability of dispersal-recruitment models and the role of dispersal limitation versus microsite limitation have not been examined for specialized habitats. In patchy, freshwater *Myristica* swamp forests (Western Ghats, India), we examine the roles of primary seed dispersal, secondary seed removal and microsite suitability for the establishment of a swamp specialist tree, *Myristica fatua*. We estimated primary seed shadows, performed secondary removal experiments and enumerated recruits in swamp sites. Steady-state fruiting was observed with the extended production (>7 months) of small numbers of fruits. Frugivores dropped most of the large and heavy seeds under parent crowns, while a few seeds were transported over short distances by hornbills. Seed placement experiments indicated that removal, germination and establishment were similar within swamp microsites, while seeds failed to survive in matrix habitats surrounding the swamp. Crabs, which were major secondary removers of *M. fatua*, did not alter the initial seed dispersal patterns substantially, which led to the retention of seeds within the swamp. Distribution of saplings and adults from previous seasons also suggest that dispersal-recruitment dynamics in the swamp specialist *M. fatua* did not strictly follow predictions of Janzen-Connell model while abiotic effects were significant. Large seeds, steady-state fruiting and small crop sizes may be significant selective forces facilitating escape from density and distance-dependent effects in space and time in specialist plant species such as *M. fatua*.

## Abstract (precis)

Species restricted to specialized, rare habitats such as *Myristica fatua* cannot afford to send propagules too far and risk arriving in inhospitable habitats. We followed the fate of seeds from fruiting till seedling establishment to examine ecological strategies such species employ to escape from seed predators and find the right germination sites. We found that *M. fatua* bears few large-sized seeds and fruits for extended periods of time such that few seeds are produced at any point of time, thus escaping detection of seed predators. Hornbills and crabs facilitate this by dispersing heavy seeds only to short distances within swamps.

## Introduction

A multitude of biotic and abiotic factors are known to act on different stages in the lifecycle of plants starting from seed dispersal to seedling establishment (Herrera *et al.* 1994; Howe and Smallwood 1982; Nathan and Muller-landau 2000; Wang and Smith 2002). Though their importance is likely to vary with species and habitats, current theories of seed dispersal patterns and post-dispersal processes have emerged largely from habitats that are widely distributed at regional and global scales. On the other end, habitats can be extremely specialized and encompass species with strong habitat associations. Examples of such terrestrial habitats include calcareous grasslands, coastal mangroves, heathland forests, high-altitude forests, rocky cliffs, swamp forests etc. Specialized habitats are analogous to natural habitat- islands amidst contrasting surrounding matrix, especially for taxa that are restricted to these habitats. These habitats are intrinsically different as they are often rare at regional scales, small in size with high inter-patch isolation, and have special abiotic conditions. Plant species restricted to such specialized habitats face the challenge of dispersing their propagules within these habitats and limiting dispersal to unfavourable surrounding habitats (Bazzaz 1991). Thus, ecological processes such as seed dispersal and recruitment of these plant species are of great interest to understand evolutionary strategies that facilitate their persistence in these habitats. Generally speaking, such studies are lacking in these habitats with a few exceptions (e.g. Clarke *et al.* 2001; Garcia *et al.* 2009; Rand 2000).

Habitat specialization can act as a strong filter determining the fate of seeds and spatial patterns of recruitment (Lin *et al.* 2013; Palmiotto *et al.* 2004). As a result, general predictions that apply to the dispersal-recruitment dynamics of habitat generalist plant species, may be invalid or upstaged by other factors. One such extensively tested prediction, the Janzen-Connell (JC) model, explains the density and distance-dependent effects of proximity to conspecifics on recruitment in widespread habitats (Connell 1971; Janzen 1970). Dispersing seeds away from the parent plants and other high-density seed deposition areas, can lead to escape in space from the action of density and distance-dependent enemies. A recent meta-analysis of 108 plant species, suggests overall support for JC effects in both tropical and temperate regions (Comita *et al.* 2014). Thus, ‘escape in space’ from JC effects via dispersal is a well-explored theme in habitat generalists (Beattie and Lyons 1975; Hay and Fuller 1981; Janzen 1972). Another way to avoid these effects is by regulating the numbers of fruits overtime and the timing of fruit production. Surprisingly, ‘escape in time’ has been little studied in general; although a few studies have addressed it in the context of flowering, in which extended flowering or mass flowering may reduce pre-dispersal seed predation (Albrectsen 2000; Atlan *et al.* 2010; Molau *et al.* 1989).

Against this background, we examined natural seed rain patterns as well as followed fates of seeds close to and away from conspecific trees in a habitat specialist tree, *Myristica fatua var. magnifica*, which occurs exclusively in the narrowly distributed *Myristica* swamp forests, restricted to the Western Ghats in India. Naturally small and patchy, the *Myristica* swamps occur in a few confined valleys along streams flowing through evergreen, semi-evergreen or moist deciduous forests (Chandran *et al.* 1999; Roby *et al.* 2014). These forests lie interspersed in a matrix of non-swamp forests. Owing to its extreme habitat specialization, *M. fatua* is a suitable candidate tree species to examine strategies that permit persistence in this specialized habitat, and whether broad generalizations that are applied to the dispersal-recruitment dynamics of widespread habitats and generalist species can be redeployed to a narrowly distributed habitat specialist species.

Here, our goal is to understand dispersal-recruitment dynamics in this swamp specialist species by examining seed rain patterns in conjunction with post-dispersal seed fate and suitability of abiotic conditions. We used a forward approach, following seeds from their production, movement from parent trees up to their establishment along with the backward approach of looking at distribution of saplings and inferring the processes that shape these patterns. Using this integrated approach in a combination of experimental and observational methods, we attempt to understand the spatiotemporal strategies that allow persistence in this specialized habitat by asking the following questions:

- What is the spatial pattern of seed rain in *M. fatua*?
- Does secondary seed removal and recruitment *of exper*imentally placed seeds vary in different microsites and with proximity to fruiting trees?
- Does the distribution of saplings suggest th*e presen*ce of density- and distance-dependent effects for establishment in *M. fatua*?

## Methods

### Study system and species

*M. fatua* is an endangered, endemic canopy tree species (height = 20–25 m) (IUCN 2000; Ramesh *et al.* 1997). Trees are dioecious and flower from December to April. *Myristica* swamp forests, dominated by Myristicaceae members, are distinctive lowland communities exclusive to the Western Ghats of India. The swamps differ in their vegetational composition and soil properties from the surrounding matrix and the edge of the swamp (2–5 m wide), which is a raised slope forming a sharp ecotone and resulting in distinctive microsites (Vijayakumar and Vasudeva 2011). The best preserved representatives of this habitat are found in our study region, i.e. Kulathupuzha reserve forest (latitude 8° 51 N, longitude 77° 5 E) in the southern Western Ghats in India **[see Supporting Information Fig. 1]**. Swamp sizes are typically small, ranging from 0.1 to 20 ha.

Our study was conducted in three relatively undisturbed swamp patches. These include, two small swamp patches (1.5 ha each), namely, Marappalam (hereafter S1), Pullumala (hereafter S2) and the third is a relatively large swamp, Munnamchal (hereafter L1, >20 ha). Key structural characteristics of the swamp patches such as width of the swamp, adult tree densities, tree species richness, % canopy cover and clustering of *M. fatua* adult trees are presented in Table 1 **[see Supporting Information** for methodological details and analyses and Table S1**]**. Sapling distribution was recorded in five additional swamps **[see Supporting Information]** apart from S1, S2 and L1 i.e. Ambalathupacha (hereafter S3, 2.5 ha), Valiyapacha (hereafter S4, 1 ha), Chettadi (hereafter L2, 8 ha), Dalikarikkam (hereafter L3, 9.6 ha) and Neerattuthadam (hereafter L4, 16 ha).

**Table 1.** Key characteristics of the study swamps S1, S2 and L1 are shown. Differences among study swamps in tree densities (/ha), species richness, percent canopy coverage, width of the swamp and nearest neighbour distances between adult *M. fatua* trees are presented with their corresponding chi-square values (df = 2). The table gives spatial distribution patterns of adult *M. fatua* trees characterized using Clark and Evans dispersion index (R). ‘*’indicates statistical significance and significant pairwise differences between swamps are shown with different letters (pairs a-a indicate no significant difference and pairs a-b indicate significant difference between swamps).

	**S1**	**S2**	**L1**	***χ*^2^**	***P*-value**
Tree density (/ha)	720	816	779	1.06	0.58
Rarefied species richness	20.13	14.76	24.57	3.03	0.21
Canopy coverage (%)	87.11	84.88	89.32	1.06	0.58
Nearest neighbor distances (m)	3.97 ±10.66**^*a*^**	4.07 ±4.15**^*a*^**	10.12 ±10.06**^*b*^**	15.90	<0.001*
Width of the swamp (m)	30.44 ±12.15**^*a*^**	38.07 ±11.33**^*a*^**	63.04 ±33.76**^*b*^**	7.95	0.01*
**Clark and Evans dispersion index (R)**					
Adult *M. fatua* trees	0.47	0.47	0.41	0.009	0.99

### Spatial patterns of seed rain

#### Fruiting phenology and seed size

**Figure 1.**
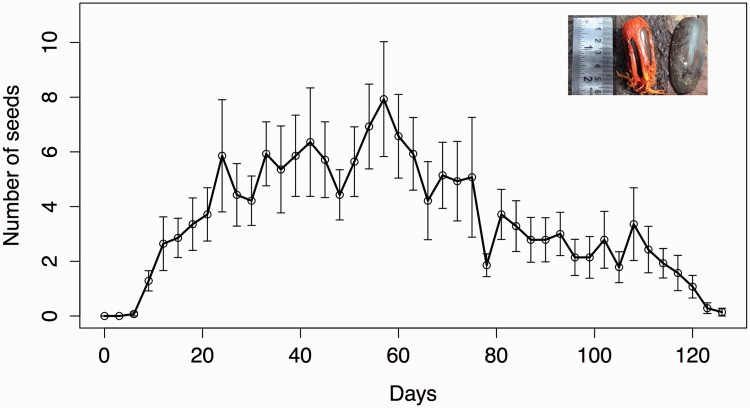
Number of seeds dispersed via frugivores and those passively detached from *M. fatua* trees over the fruiting months (28 May to 31 September, 2013). The error bars indicate SE values. Inset shows an aril-intact seed (non-dispersed) and an aril-removed (dispersed) seed.

Capsules are large, hard and dehisce partially while still attached to the tree exposing the bright reddish-orange aril that covers a single large oblong seed (Fig. 1). Seeds that fell on the ground arrived there via two routes: (i) dispersal by frugivores that consumed the aril and dropped seeds intact (henceforth dispersed seeds), and (ii) seeds with intact arils that detached passively upon maturity (henceforth non-dispersed seeds; Fig. 1).

We monitored fruiting phenology of female trees (eight trees each in S1, S2, L1) at fortnightly intervals from January to December 2013. Trees were scored 1–4 in intervals of 25 %, based on the percentage of crown fruiting (Fournier 1974). Fresh weights and dimensions (length and width) of seeds were obtained (*n* = 30 seeds).

Additionally, we also estimated natural patterns of seed fall using methods followed in earlier studies (Caraballo-Ortiz *et al.* 2011; Howe 1983) by counting the number of capsules under fruiting crowns (eight trees in S1 and six trees in S2). Crown areas of these female trees ranged from 6.12 to 54.47 sq. m. Capsule counts gave accurate estimates of seed fall over the fruiting season as well as crop sizes of trees, since the inedible capsules were not removed. Numbers of capsules, dispersed (aril-removed) and non-dispersed (aril-intact) seeds under fruiting crowns were counted every third day for 130 days from the beginning of fruit maturation in May.

#### Primary frugivory

Preliminary observations in swamps S1, S2 and L1 showed that Malabar grey hornbill (MGH) (*Ocyceros griseus*) and bonnet macaque (*Macaca radiata*) removed most *M. fatua* fruits, though these frugivores did not swallow or regurgitate the large seeds. A small proportion of fruiting crop was also removed by Nilgiri langurs (*Trachypithecus johnii*), lion-tailed macaques (*Macaca silenus*) and Malabar giant squirrels (*Ratufa indica*) occassionally. Hornbills ate the aril on the parent tree and dropped the seed beneath trees or rarely flew away with a seed to process it perched short distances away. Macaques on the other hand always consumed the aril and dropped the seed under parent trees.

We observed frugivore activity in the mornings (0700–1100 h) and afternoons (1200–1600 h) within a 1.5 ha area in S1, S2 and L1 swamp sites (total of 384 h over 24 days in each swamp) alternating between the sites on a daily basis. Observations were conducted by two observers at six randomly chosen fruiting *M. fatua* trees in each swamp to record the microsites of seed deposition by frugivores. Additionally, no nocturnal frugivores were detected at fruiting trees during observations between 1900 and 2300 h in S1 over 10 nights using night vision binoculars. During the day, once a frugivore was seen holding a fruit, it was observed till the seed was dropped and the distance from the source tree was recorded. Focal trees were typically located towards the swamp interior and as one move away from the interior of the swamp, variations in inundation characteristics resulted in varied microenvironments. Based on these observations, we identified the following zones as microsites (i) under parent tree crowns (0 m), (ii) away from conspecific crowns within the swamp (3–8 m from the parent crowns), (iiii) edge of the swamp and (iv) matrix (region surrounding the swamp). In order to analyze differential seed deposition in these microsite types we used a generalized linear mixed model with Poisson error distribution (number of seeds deposited) including microsite as fixed effect and focal tree identities nested within swamp as random effects in lme4 package (Bates *et al.* 2015). A Tukey’s test for *post hoc* pairwise comparisons was conducted using the glht() function in the multcomp package (Hothorn *et al.* 2008).

### Secondary seed removal

In our previous study, we found that secondarily removed seeds met with mixed fates in which crabs secondarily removed >60 % of *M. fatua* seeds (moved seeds into burrows which facilitated escape from predation), while 25 % of the seeds were subjected to predation by rodents and squirrels or weevil infestation (1 %) (Krishna and Somanathan 2014).

To quantify secondary removal, we experimentally placed dispersed seeds (aril-removed) in the four microsites described above (a–d) into which frugivores were observed to drop seeds. At eight equidistant points (35 m apart) along the main stream in each of the three swamps, four seed stations (7–15 m apart, three seeds/seed station) were established in each of these four microsites (eight seed stations/microsite). Such seed additions allowed us to control for any variation in the age of seeds, aril to seed mass ratio, aril colouration and odours that may bias removal by animals. Distance from each seed station to the nearest conspecific fruiting tree was recorded along with diameter at breast height (DBH) of fruiting trees within 5 m of the seed station. Since the density of seeds in the neighbourhood is expected to increase with increasing size of the trees and decrease with increasing distance to the seed station, we used a proximity index which is a modification of neighborhood competition index (Canham 2004). This allowed us to understand the role of distance and density-dependent effects at the seed stage. Seed removal from these stations was monitored on Days 1, 2, 3, 4, 5, 7, 9, 14 and weekly thereafter for 12 weeks. To compare seed removal rates as a function of proximity to fruiting conspecifics and across different microsites we used a Cox proportional hazards model with mixed-effects in the R package coxme (Therneau 2012). In this model, microsite and proximity index were included as fixed effects, and seed stations nested within swamp, were included as random effects.

As crabs were known to be the major secondary seed removers, we estimated the densities of crab burrows within 5 × 5 m plots (32 plots/swamp, in the three study swamps) with seed stations as the centre, both under the female tree crowns as well as away from crowns.

### Seedling establishment

Recruitment of seedlings in different microsites was examined by placing seeds in aluminum mesh exclosures to prevent seed removers. Eight exclosures (mesh size = 1.03 mm, dimensions = 0.3 × 0.3 × 0.5 m) were placed in each of the four microsite categories in the large swamp, L1 (three seeds/exclosure, eight exclosures/microsite). Also, DBH of fruiting trees within 5 m of the exclosures was recorded. Open seed stations in the site L1 from the previous experiment served as controls for those placed inside the exclosures. Seed germination and seedling establishment was monitored at fortnightly intervals from August till the end of December 2013. Generalized linear mixed models (glmer function in R) with binomial error distributions were used to analyze germination and seedling establishment with microsites and basal area of trees around as fixed effects along with incorporating exclosure identity as a random effect to account for replicates nested within each microsite.

### Sapling distribution

We inferred density- and distance-dependent survival beyond the seedling stage from previous seasons’ crop by recording the number of *M. fatua* saplings (height = 0–5 m) under parent crowns and away from crowns (nearest female tree at least 5 m away) in the three main study swamps (S1, S2, L1) and in five additional *Myristica* swamp patches (S3, S4, L2, L3 and L4; **[Supporting information Table S1]**). Saplings recorded away from crowns included those in the swamp interior and edge. In four of the small swamps (1–3 ha, S1–S4), number of saplings, number of female trees and their DBH were recorded for the entire swamp. In the remaining four large swamps (10–20 ha, L1–L4), number of female trees with their DBH and number of saplings were sampled in twenty plots (dimension 20 × 20 m). Female tree sizes were used to calculate mean basal areas (m^2^/ha) for each of the swamps, to provide an estimate of variation in seed production across the swamps. Differences in sapling densities were analyzed using linear mixed effects model (lmer function in R) including microsite (under and away from crowns) as fixed effect and female tree densities as random effect to control for the influence of number of female trees within each of the swamps.

All statistical analyses were conducted using R software (R Development Team 2013). Results are presented as mean ± SD unless otherwise stated. For all generalized linear mixed effects models (GLMMs) performed, a likelihood ratio test (chi-square) comparing the full model with a reduced model including the random effects but without the fixed effects term was used.

## Results

### Spatial patterns of seed rain

#### Fruiting phenology and seed size

Phenology of *M. fatua* is typical of a steady-state species with extended and low intensity fruiting (March–November). More than 90 % of individuals fruited for over 6 months. Trees produced 38–315 fruits in a fruiting season (*n* = 14). Seeds were large and heavy (weight= 20.9 ± 2.93 g, length = 50.25 ± 4.2 mm, width= 22.1 ± 3.5 mm, *n* = 30 seeds) with little variation between trees. The average number of fruits removed was < 3 per tree per day (Fig. 1).

#### Primary frugivory

MGHs dispersed 65.9 % of the seeds (*n = *364 seeds) while other frugivores dispersed the remaining seeds. In the large swamp L1, the observed seed rain was largely due to hornbills since visits by other frugivores were rare (Pairwise Wilcoxon rank sum test, S1 and S2–L1, *P* < 0.001, S1–S2, *P* = 0.95, Table 2).

**Table 2.** A summary of primary seed dispersal patterns in the study swamp swamps, S1, S2 and L1. Differences among study swamps in seed dispersal distances, seeds dispersed by hornbills (MGH), seeds dispersed per observation session (mean ± SE) and seed deposition into different microhabitats are shown with their corresponding chi-square values (df* = *2). ‘*’indicates statistical significance and significant pairwise differences between swamps are shown with different letters (pairs a-a indicate no significant difference and pairs a-b indicate significant difference between swamps).

	**S1**	**S2**	**L1**	***χ*^2^**	***P*-value**
Seeds dispersed/session	6.87 ± 3.46	5.86 ±3.29	3.5 ±2.12	0.04	0.97
Dispersal distance (m)	4.95 ± 0.55**^*a*^**	8.84 ±1.32**^*b*^**	6.84 ±1.36**^*a*^**	0.026	0.01*
Seeds dispersed by MGH (%)	60.90**^*a*^**	52.89**^*a*^**	83.96**^*b*^**	23.16	<0.001*
Seed rain under crowns (%)	61.02**^*a*^**	57.98**^*a*^**	40.88**^*b*^**	9.47	0.008*
Seed rain away from crowns (%)	9.57	8.24	19.19	6.60	0.03*
Seed rain in the edge (%)	6.3	7.79	3.64	1.58	0.45
Seed rain in the matrix (%)	23.1	25.97	36.27	4.71	0.09

**Figure 2.**
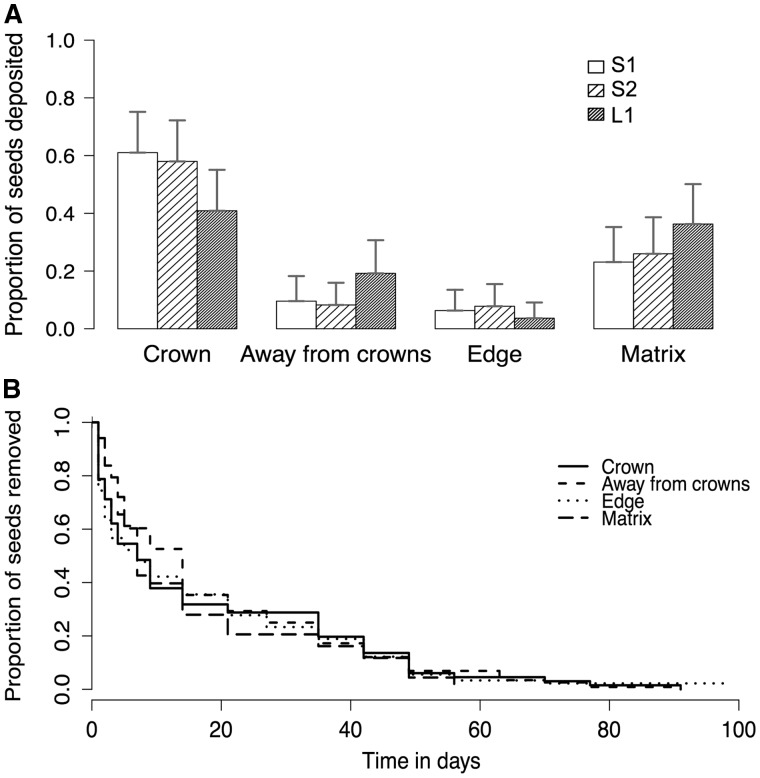
**(A)** Proportion of seeds dispersed by frugivores in the different microsites i.e. crown, away from conspecific crowns, edge of the swamp, and surrounding matrix in the three study swamps, S1, S2 and L1; bars represent mean (+SE) and **(B)** Secondary removal rates of dispersed (aril-removed) seeds from different microsites where seed deposition was observed.

Most seed dispersal was highly localized with dispersal distances in the three study swamps ranging from 0 to 15 m (Table 2). Seed deposition was the highest under crowns (Table 2, Fig. 2A). A large percentage of seeds dispersed by frugivores landed either under crowns of parent trees or in the matrix surrounding the swamp while the rest were deposited away from crowns but within the swamp or in the edge (Table 2). Microsite type had a significant effect on the pattern of seed dispersal (likelihood ratio test, *χ*^2^= 210.72, df = 3, *P*0.001) with most of the seeds reaching crown and matrix microsites. Proportion of seeds deposited under the crowns differed significantly from all the other three microsites i.e. away from crowns, edge and matrix (Tukey’s contrasts, *P* < 0.001, Table 3).

**Table 3.** Parameter estimates for pairwise comparisons (Tukey’s contrasts) of differential seed deposition in different microsites included in (poisson GLMM) and influence of microsites and proximity index on secondary seed removal of Cox regression mixed-effects model. Model estimates, standard errors (SE), *z*-values (z) and *P*-values are presented.

Parameter	Estimate	SE	*z*	*P*-value
Primary frugivory model (microsite as fixed factor and tree identity nested within swamp as random factors)				
Crown-away	1.6447	0.1730	9.504	<0.001
Edge-away	−0.5281	0.2601	−2.030	0.167
Matrix-away	0.7553	0.1921	3.932	<0.001
Edge-crown	−2.1728	0.2177	−9.979	<0.001
Matrix-edge	−0.8894	0.1290	−6.895	<0.001
Matrix-crown	1.2833	0.2331	5.505	<0.001
Secondary removal model with crown microsite as reference group (microsite and proximity index as fixed factors and seed station nested within swamp as random factors)				
Away from crown	0.0269	0.2016	0.13	0.89
Edge	−0.0244	0.2064	−0.12	0.91
Matrix	0.0621	0.2295	0.27	0.79
Proximity index	0.0002	0.0005	0.39	0.69

### Secondary seed removal

In seed placement experiments, 90–100 % of seeds were secondarily removed within 98 days of exposure in the three study sites. Interestingly, secondary seed removal rates were similar between the different microsites (Cox regression-likelihood ratio test, *χ*^2^ = 0.258, df = 4, *P* = 0.99, Fig. 2B, Table 3). Seed stations in different microsites varied in their proximity to conspecific fruiting trees from 0 to 19.95 m. However, proximity and size of the fruiting trees (z = 0.39, *P = *0.69) did not affect secondary seed removal rates. Furthermore, the densities of major secondary seed removers of *M. fatua*, the crab (*Barytelphusa guerini*), are similar under (0.320 ± 0.169) and away from female tree crowns (0.317 ± 0.167) in all the three swamps S1, S2 and L1 (Wilcoxon rank sum test, *W* = 126, *n* = 32, *P* 0.05).

### Seedling establishment and sapling distribution patterns

#### Germination and seedling establishment in different microsites

Secondary removal of control open seeds placed adjacent to exclosures was very high with >85 % removal, while this was prevented for seeds within exclosures, indicating that the exclosures were effective. Germination and establishment of seeds placed within mesh exclosures was strongly influenced by the microsite type (Binomial GLMM- likelihood ratio test, *χ*^2 ^=19.63, df = 4, *P* < 0.001; establishment, *χ*^2 ^=10.09, df = 4, *P* = 0.03) while basal area of fruiting trees had no effect (*z* = 0.081, *P* = 0.93). The proportion of seeds that germinated was significantly higher under crowns, away from crowns and edge compared with matrix microsites (Tukey’s contrasts, *P* < 0.05) suggesting that most parts of the swamp are suitable for recruitment. The percentage of germinated seeds that established declined uniformly over two months (Kruskal-Wallis test, *χ*^2^ = ^ ^40.1, df = 1, *P* < 0.001) in all the microsites and none of the germinated seeds survived in the matrix.

#### Sapling distribution

**Figure 3.**
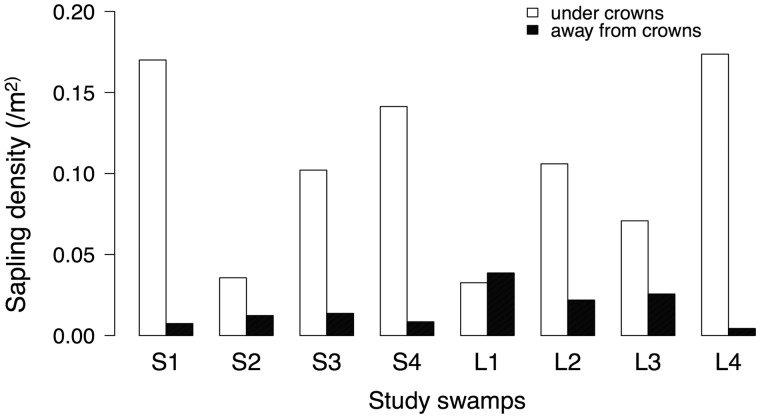
Bar chart shows a comparison of *M. fatua* sapling densities (/m^2^) under the crowns versus away from crowns in eight *Myristica* swamp patches. Swamp sites, L1, L2, L3, L4 are large swamps while S1–S4 are small swamps.

The density of established saplings from previous seasons was significantly higher under the crown versus away from crowns in S1 (*χ*^2 ^=144.32, df =1, *P* 0.001) and S2 (*χ*^2 ^=^ ^27.16, df =1, *P* 0.001) sites, while in L1 similar densities were found (*χ*^2 ^=1.48, df = 1, *P* = 0.223). Mean basal area of fruiting trees for large swamps, L1–L4, ranged from 1.60 to 4.237 m^2^/ha while for the small swamps, S1–S4, it ranged from 0.84 to 4.65 m^2^/ha. In seven of the eight *Myristica* swamps (three main and five additional sites), areas away from the crown had 60–98 % lower sapling densities than under crowns and thus sapling densities were 6-fold higher under crowns (Binomial GLMM-likelihood ratio test, *χ*^2 ^=13.78, df =1, *P* < 0.001) (Fig. 3). Adult densities of the eight study swamps were comparable ranging from 0.001 to 0.003/m^2^.

## Discussion

Dispersal-recruitment dynamics in *M. fatua*, a swamp specialist tree, did not follow the predictions of the JC model for seed survival and recruitment. We argue that escape in space and time from density dependent effects may be facilitated by a suite of fruiting traits, which together can influence the behaviour of primary frugivores and secondary removers.

### Fruiting patterns, frugivore behaviour and seed dispersal patterns

*M. fatua* trees fruited for an extended period of 7–9 months and had small crop sizes (38–315 fruits), resulting in low rates of seed fall (3 per day/tree). Extended flowering which is commonly considered a strategy to reduce the risk of reproductive failure (Bawa 1983; Elzinga *et al.* 2007; Rathcke and Lacey 1985; Tarayre *et al.* 2007) leads to asynchronous and low rates of fruit maturation which in turn can impact pre-and post-dispersal seed predation. Seed predation can shape flowering and consequently fruiting schedules via different mechanisms: (i) flowering and fruiting out of phase with seed predators and (ii) minimizing detection by seed predators by maintaining a low and steady level of fruit production (‘steady-state strategy’, Albrectsen 2000; Crawley 2000; Gentry 1974; van Schaik *et al.* 1993) due to which seed fall is gradual and minimal or alternatively by producing massive fruit numbers in a short, big burst (‘big bang strategy’), thus potentially satiating seed predators (Forget 1992; Janzen 1971; Stevenson *et al.* 2005). Of these, the predator satiation hypothesis as a temporal escape strategy has been studied extensively in the context of masting in which a very large number of fruits are produced synchronously at irregular intervals (Curran and Webb 2000; Forget 1993; Takeuchi and Nakashizuka 2007). On the other hand, extended fruiting as a possible ‘escape in time’ strategy has never been discussed before in the context of post-dispersal seed predation.

Fruits and seeds of *M. fatua* are exceptionally heavy and are therefore transported by frugivores only over small spatial scales. Though large-bodied vertebrates typically forage over large ranges, heavy seeds are usually dropped close to parent crowns especially when seeds are inedible (Clark *et al.* 2005; Lambert and Chapman 2005; Spiegel and Nathan 2007). Although, birds can contribute to long-distance dispersal in many species (Carlo *et al.* 2013; Holbrook and Smith 2000; Speigel and Nathan 2007), in our study, we consider this a rare event as hornbills consumed the lipid-rich arils on the parent tree and dropped the large seeds under crowns mostly, resulting in highly localized seed shadows. By connecting the observed seed dispersal pattern to seedling establishment in the different microsites, we found that >70 % of seeds reached suitable microsites within the swamp via dispersal by frugivores. Occasional flights by hornbills carrying an arillate seed resulted in seeds being dropped in the matrix. Seed transport over distances >15 m (mean distance = 7.18 ± 3.50 m) enhanced the chance of their deposition in unsuitable non-swamp habitat where lack of hypocotyl elongation followed by propogule death was noted within three weeks. Adult trees or saplings were also absent in the matrix habitat indicating a sharp ecotone for abiotic conditions between the swamp and the surrounding habitat. Thus, we hypothesize that there could be selection for large seed size and extended fruiting in *M. fatua* which have a bearing on the behaviour of primary frugivores, resulting in localized seed shadows and possibly maintaining seed densities below the threshold of density-dependent effects. This remains to be tested in other swamp specialist species that differ in seed traits and fruiting phenology.

### Post-dispersal seed fate in different seed deposition microsites

Animals are known to respond to microsite types such as edges, canopy and gaps resulting in differential secondary seed removal patterns (Chauvet and Forget 2005; Ries *et al.* 2004). Ants, dung beetles and scatterhoarding rodents are known to alter primary seed shadows substantially in other habitats (Feer 1999; Forget and Milleron 1991; Roberts and Heithaus 1986; Roth and Vander Wall 2005; Wenny 1999). However, in our study, secondary agents evenly removed seeds in all microsite types (>90 % of total seeds in seed stations). The localized dispersal by crabs (mean distance = 1.89 m) into or close to burrows to consume the arils can preclude seed predation by other agents as well as retain seeds within the swamp habitat without substantially altering the initial spatial pattern of seed deposition (Krishna and Somanathan 2014).

Thus, the templates generated by biotic (primary and secondary removal) agents overlapped significantly with the abiotic conditions that favour recruitment in *M. fatua*. In most species, requirements for seedling establishment and survival are different from those found in high seed arrival zones (Augspurger 1984; Jordano and Herrera 1995; Spiegel and Nathan 2011). Such conflicting requirements between life stages are common and driven by biotic factors, resulting in the recruitment patterns predicted by the JC model (Herrera *et al.* 1994; Horvitz and Schemske 1994; Rey and Alcantara 2000; Schupp 1995). Recruitment limitation in habitat specialist species is reported primarily due to restrictions imposed by special abiotic conditions such as in mangrove species and chiltepin species, which are associated with specific nurse trees (Carlo and Tewksbury 2014; Clarke *et al.* 2001). Our findings reinforce the notion that recruitment in specialist species such as *M. fatua* are more likely to be limited by abiotic conditions than by dispersal. In such species, the probability of a seed being dispersed is much higher than it germinating in the dispersed microsite (Moore and Elmendorf 2006; Münzbergova and Herben 2005).

A synthesis of several studies investigating density and distance-dependent effects on seed and seedling establishment has largely illustrated compliance with the predictions of the JC model, while deviations are rare (Comita *et al.* 2014). These deviations have been attributed to predator satiation resulting from high seed densities during masting, foraging patterns of vertebrate and invertebrate seed predators, better suited abiotic conditions closer to parent trees and chemical defenses in seeds (Bustamante and Simonetti 2000; Condit *et al.* 1992; Forget 1993; Norghauer *et al.* 2006; Notman *et al.* 1996; Takeuchi and Nakashizuka 2007). In *M. fatua*, seed deposition and establishment beneath fruiting crowns and the clustered distribution of adult trees points to a lack of density and distance-dependent effects. High overlap in deposition and establishment microsites suggests highly suitable abiotic conditions in proximity to adults along with possible spatio-temporal strategies for evading the negative effects of proximity to conspecific trees.

We found that distance to conspecific fruiting trees and their size (crop size) did not influence secondary seed removal rates significantly. The uniform distribution of crab burrows in the swamp habitat, their territoriality and short-distance foraging could have resulted in the observed short distance seed removal patterns. High sapling abundance around female tree crowns also suggests lack of density and distance-dependent seed predation and seedling herbivory. Thus, we find that the JC model, which predicts high seed and seedling mortality near conspecific trees, is unlikely to be applicable to *M. fatua.* Higher sapling densities under female tree crowns than away from them also provide supportive evidence towards this.

### Significance of escape in space and time

Escape in space from density-dependent effects is often considered, while the role of temporal escape is largely ignored. Temporal escape has been discussed only in the context of pre-dispersal seed predation so far, where extended or late flowering plants escape from high levels of predation (Albrectsen 2000; Atlan *et al.* 2010). Overall, we attribute the observed non-compliance with the JC predictions in *M. fatua* to the extended production of a few, large-seeded fruits which result in low seed densities on the forest floor. Although the limited availability of suitable habitat within the swamp can result in crowding of seeds on the forest floor and potentially attract density-dependent enemies, the extended period of fruiting can provide a means to minimize the negative effects of competition with conspecifics while escaping detection by density-dependent enemies. Thus, in addition to escaping from predation in space, dispersing few seeds over an extended period suggests the significance of escape of seeds in time in *M. fatua*. Ongoing studies on fruiting phenology over 4 years (2011–15) at these sites suggest that inter-annual variation in fruiting intensities is minimal (unpubl. data). However, we emphasize the need to consider spatio-temporal variations in longer term studies as well as integrating ecological and genetic data to examine the role of seed predation as a selective force in the evolution of fitness-related traits in *M. fatua* and in other habitat specialists.

## Conclusions

In *M. fatua*, we found that abiotic factors exert a relatively higher control over establishment and distribution patterns than biotic factors. Thus, we conclude that spatially-explicit models of seed dispersal as well as influence of biotic agents on the subsequent fate of seeds that have been extensively examined in widely distributed species might not be entirely valid for specialist species such as *M. fatua*. Although *M. fatua* provides probably the first case, further research is required to explore the diversity of strategies habitat specialists employ to survive in their specialized habitats.

## Sources of Funding

This study was supported by funding from Indian Institute of Science Education and Research Thiruvananthapuram and from the Department of Science and Technology, India.

## Contributions by the Authors

H.S. conceived the study; H.S. and S.K. designed the experiments and wrote the article; S.K. carried out the experiments and analyzed the data.

## Conflicts of Interest Statement

None declared.

## Supplementary Material

Supplementary Data

## Supporting Information

The following additional information is available in the online version of this article—

**File 1.** Map of the Western Ghats in India, showing the study region along with a schematic of study sites and a brief description of habitat structure of the three main study sites.

Table S1. Summary of study sites.
