# Supplementary material for: Spatiotemporal strategies that facilitate recruitment in a habitat specialist tree species
Source: AoB Plants. 2016 Jul 11;8:plw033. doi: 10.1093/aobpla/plw033 (PMC4940510; doi:10.1093/aobpla/plw033)
Supplement: Supplementary Data [file supp_8_plw033_index.html]

Spatiotemporal strategies that facilitate recruitment in a habitat specialist tree species — Supplementary Data 

# Spatiotemporal strategies that facilitate recruitment in a habitat specialist tree species

## Supplementary Data

files

- Supplementary Data - docx file
