# Supplementary material for: Spatiotemporal strategies that facilitate recruitment in a habitat specialist tree species
Source: AoB Plants. 2016 Jul 11;8:plw033. doi: 10.1093/aobpla/plw033 (PMC4940510; doi:10.1093/aobpla/plw033)
Supplement: Supplementary Data [file supp_plw033_aobplants-15319-s01.docx]

**SUPPORTING INFORMATION**

**Map of the Western Ghats in India, showing the study region and a schematic of study sites**

**
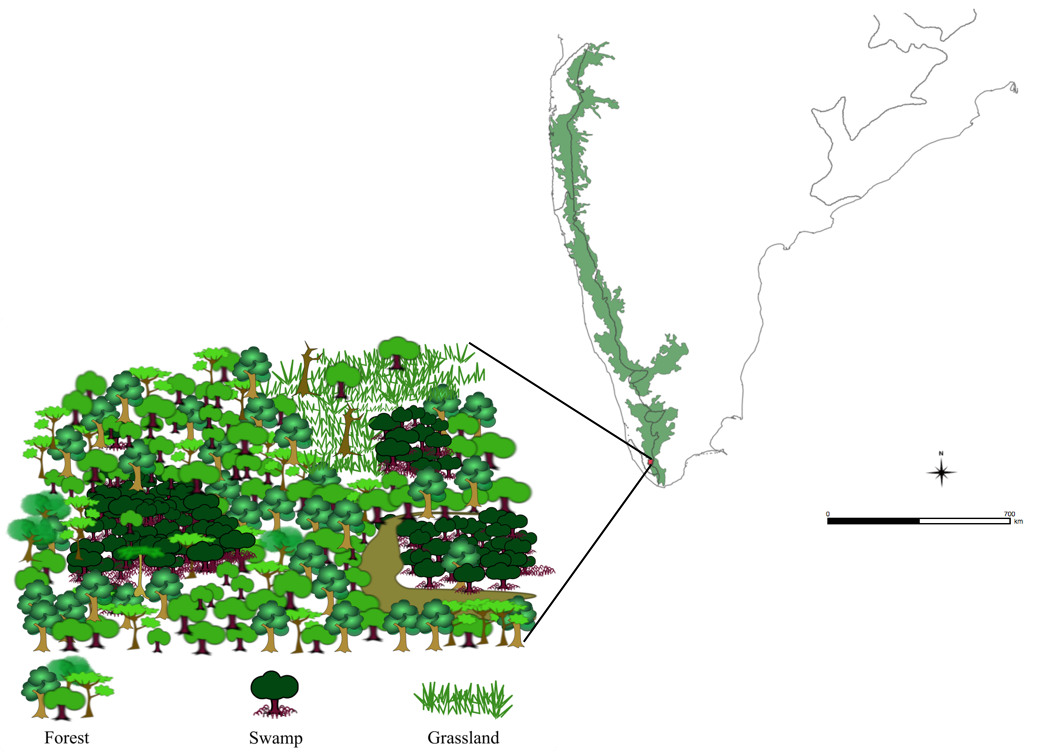
**

**Description of habitat structure of the three study sites**

Site-specific differences in structural characteristics including width of the swamp, adult tree densities, tree species richness, and % canopy cover were considered to understand differential seed dispersal patterns. From the mapped areas of the swamps, we extracted width of the swamp from side-to-side measurements at 15 randomly chosen points. In six 400 sq. m plots, identity of trees species and canopy cover were recorded. Multiple Kruskal-Wallis tests were used to compare structural differences between study sites and Wilcoxon rank sum post-hoc test with Bonferroni corrections was used for pairwise comparisons between swamps. All adult *Myristica fatua* trees in the two small study swamps S1 and S2 were mapped, while adults within a 1.5 ha area were mapped in the large swamp L1. Clustering of these trees was estimated using Clark and Evans aggregation index (R) in the R package ‘Spatstat’ (Baddeley and Turner 2005). A value R<1 indicates clustering while R>1 indicates a regular pattern (Clark and Evan 1955).

The three study sites had comparable tree densities, canopy cover and rarefied tree species richness (Table 1). Apart from the large total area of the swamp, L1, the mean width of the swamp was significantly higher (63.04 m) than that of S1 (30.44 m, S1-L1, *P* = 0.02) while S1 and S2 were comparable in their width ranges (S1-S2, *P* = 0.42, Table 1). Clark and Evans (R) aggregation indices for the three main study swamps S1, S2 and L1 were less than 0.5, suggesting a spatially clustered pattern of adult distribution. All the values significantly deviated from random distribution at α = 0.001 level (Table 1).

**Table S1: Summary of study sites**

| **Study sites** | **Area (ha)** | **Latitude and longitude** | **Surrounding habitat** | **Tree density per sq.m**  **(in surrounding habitat)** |
| --- | --- | --- | --- | --- |
| Marappalam (S1) | 1.5 | 8°52'53.75"N  77° 5'6.48"E | Evergreen forest, semi-evergreen forest (3 sides), plantation with grassland (1 side) | 0.051 |
| Pullumala (S2) | 1.5 | 8°52'23.78"N  77° 4'49.62"E | Evergreen forest, semi-evergreen forest (3 sides), plantation with grassland (1 side) | 0.050 |
| Munnamchal (L1) | > 20 | 8°51'20.89"N  77° 5'36.46"E | Evergreen forest (4 sides) | 0.060 |
| Ambalathupacha (S3) | 2.5 | 8°52'51.46"N  76°59'14.04"E | Evergreen forest, semi-evergreen and moist deciduous forest (all 4 sides) | 0.054 |
| Valiyapacha (S4) | 1.00 | 8°54'41.41"N  76°57'38.68"E | Semi-evergreen and moist deciduous forest (2 sides), oil palm plantation (2 sides) | 0.047 |
| Chettadi (L2) | 8.00 | 8°53'6.89"N  76°59'25.02"E | Semi-evergreen and moist deciduous forest (3 sides), cropland (1 side) | 0.042 |
| Dalikarikkam (L3) | 9.60 | 8°51'39.51"N  77° 4'12.64"E | Evergreen forest and semi-evergreen forest (3 sides), cropland (1 side) | 0.056 |
| Neerattuthadam (L4) | 16.00 | 8°54'52.65"N  76°58'16.93"E | Moist deciduous forest (2 sides), oil palm and acacia plantation (1 side) | 0.052 |

**Literature cited:**

Baddeley AJ, Turner R. 2004. Spatstat: An R Package for analyzing spatial point patterns.

Clark PJ, Evans FC.1955. On some aspects of spatial pattern in biological populations. *Science* **121**: 397-398.
